# Molecular Investigation of SARS-CoV-2 Circulating in Iranian Bats Using Real-Time RT-PCR for Detection of Envelop (E) Gene of the Virus

**DOI:** 10.1155/2024/5313346

**Published:** 2024-02-01

**Authors:** Saeed Shahabi, Kourosh Azizi, Yaser Bakhshi, Neda Pirbonyeh, Afagh Moattari, Alireza Sazmand, Mostafa Omidian, Bahador Sarkari

**Affiliations:** ^1^Department of Biology and Control of Disease Vectors, School of Health, Shiraz University of Medical Sciences, Shiraz, Iran; ^2^Department of Biology, Faculty of Sciences, Shiraz University, Shiraz, Iran; ^3^Department of Bacteriology and Virology, Shiraz University of Medical Sciences, Shiraz, Iran; ^4^Department of Pathobiology, Faculty of Veterinary Medicine, Bu-Ali Sina University, Hamedan, Iran; ^5^Department of Parasitology and Mycology, School of Medicine, Shiraz University of Medical Sciences, Shiraz, Iran

## Abstract

**Background:**

The COVID-19 was first reported in 2019 to cause pneumonia in people of Wuhan, Hubei province, China, is now associated with high mortality worldwide. Phylogenetic analysis revealed that SARS-CoV-2 (2019-nCoV) is closely (88%–89% similarity) related to the coronavirus circulating in *Rhinolophus* (horseshoe bats). More than 50 bat species belonging to eight families have been reported from Iran of which five species belong to the Rhinolophidae family. So far, no study has been done on COVID-19 infection in Iranian bats.

**Aim:**

The current study was performed, for the first time, to investigate the infection of Iranian bats with SARS-CoV-2.

**Methods:**

This cross-sectional study was conducted in 2021 using 183 bat samples collected from three caves in the south (Fars province) and two caves in the northwest (Kermanshah and Kurdistan provinces) of Iran. Bats' digestive and respiratory system samples were collected from each bat of different species. The samples were evaluated by real-time PCR and by targeting a 221 bp fragment of the envelop (E) genes of SARS-CoV-2.

**Results:**

COVID-19 was detected in alimentary specimens of two of the Mediterranean horseshoe (*Rhinolophus Euryale*) bats.

**Conclusion:**

Although, based on the findings of the molecular evaluation, the infection of bats with COVID-19 was determined in this study, further studies are needed on a larger number of bats, particularly horseshoe bats, to confirm the potential infection of Iranian bats with COVID-19.

## 1. Introduction

SARS-CoV-2 belong to the Nidovirales order and the large family of Coronaviridae. These viruses occur naturally in mammals, birds, and reptiles such as snakes. Recently, some novel coronaviruses such as severe acute respiratory syndrome coronavirus (SARS-CoV), SARS-CoV-like viruses, Middle East respiratory syndrome coronavirus (MERS-CoV), and severe acute respiratory syndrome coronavirus 2 (SARS-CoV-2) have been discovered which cause respiratory, hepatic, enteric, and neurological diseases in human and many other animal species [1–4]. In 2005, SARS-CoV-like viruses were discovered in Chinese horseshoe and bent-winged bat species in China [5–7]. Furthermore, coronaviruses have been detected in different species of bat in Asia, Europe, Africa, and other parts of the world. COVID-19 was first reported in 2019 to cause pneumonia in people of Wuhan, Hubei province, China [4, 8] and is now associated with high mortality worldwide. Phylogenetic analysis revealed that SARS-CoV-2 (2019-nCoV) is closely related (88%–89% similarity) to the coronavirus circulating in *Rhinolophus* (horseshoe bats) [9–11]. Furthermore, molecular phylogenetic analyses suggest that SARS-CoV-2 may have originated from bats or bat droppings associated with contaminated materials in the market or surrounding regions in Wuhan [12].

The order Chiroptera is the second largest group of mammalian species after the order Rodentia and has been documented as a natural host of a large number of viruses, especially SARS-CoV-2. So far, 50 bat species belonging to eight families have been reported from Iran. The most common and abundant bats in Iran including *Miniopterus pallidus, Myotis blythii, Rhinopoma microphyllum*, and *Rhinolophus ferrumequinum* are belonging to the three families of Vespertilionidae, Rhinopomatidae, and Rhinolophidae [13, 14]. Rhinolophid bats are the most suitable species for coronaviruses infection. In Iran, the family Rhinolophidae includes five species of *Rhinolophus euryale, R. ferrumequinum, R. mehelyi, R. blasii*, and *R. hipposideros* [15, 16]. The absence of information about the infection of bats with SARS-CoV-2 in Iran justified this study which was performed for the first time to detect SARS-CoV-2 circulating in Iranian bats, based on molecular datasets of the full-length envelop (E) gene.

## 2. Methods

### 2.1. Study Design

**Figure 1:**
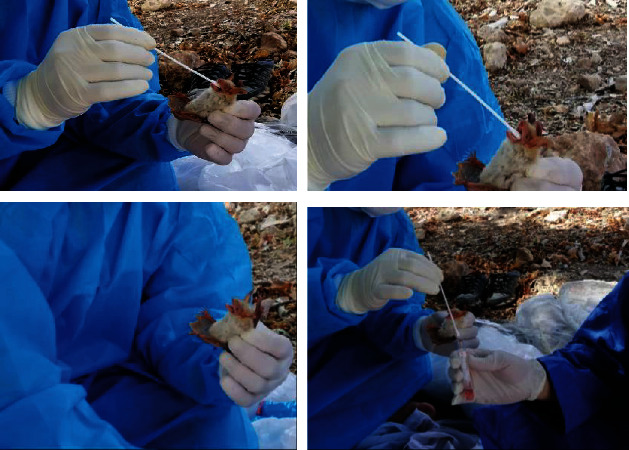
Some photos of fieldwork for collecting bats and taking respiratory samples in Iran.

This cross-sectional study was conducted in 2021 using bat samples collected from three caves in the south (Fars province) and two caves in the northwest (Kermanshah and Kurdistan provinces) of Iran (Figure 1).

### 2.2. Sample Collection

Respiratory (nasopharyngeal) and gastrointestinal (anal) swabs were collected from each bat, using sterile swabs. Using a cold box (4°C), all samples were placed in the virus transfer medium (VTM) and transferred to the Virology Department at Shiraz University of Medical Sciences, Shiraz, Iran, where these samples were kept at −20°C until use for RNA extraction. The bats were released immediately after sampling in the caves where they had been caught.

### 2.3. RNA Extraction

Viral RNA extraction was performed by using the High Pure Viral RNA Isolation Kit (Roche, Germany) according to the manufacturer's instructions. The integrity of the total RNA was determined by electrophoresis on 1% (*w*/*v*) agarose gels. Nucleic acid concentrations were measured at 260 nm. The purity of the total extracted RNA was determined as the 260 nm : 280 nm ratio with expected values between 1.8 and 2, and the extracted RNA was kept at −70°C until use.

### 2.4. Real-Time RT-PCR

COVID-19 was detected by targeting the E gene of envelop protein, using one-step real-time RT-PCR analysis (using SuperScript™ III Platinum™ SYBR™ Green One-Step qRT-PCR (Invitrogen)) on Rotor-Gene® 6000 Thermocycler (Corbett Research). The 5′-ACAGGTACGTTAATAGTTAATAGCGT-3′ and 5′-ATATTGCAGCAGTACGCACACA-3′ primers were used [17]. All oligonucleotides were synthesized and provided by Metabion (Germany). To make a 10 *μ*M working solution of primer, the 100 *μ*M stock solution was diluted (1 : 10) with sterile ddH_2_O. The RT-PCR reaction contained 6 *μ*l of 5x buffer, 1 *μ*l of enzyme mix, 1 *μ*l (10 *μ*M) of each primer, and 5 *μ*l of RNA in a total reaction volume of 30 *μ*l. RT-qPCR cycling was performed as follows: 50°C for 20 min, 95°C for 3 min, then 40 cycles of denaturation at 95°C for 10 s, primer annealing at 58°C for the 40 s, and extension at 72°C for 30 s. The fluorescence was measured at the end of each cycle. A melting curve analysis was performed following amplification to verify the specificity of the amplified products. It is consisted of 70°C for 15 s, followed by a temperature increase to 95°C for 15 s at the rate of 1.25°C/s with continuous reading of fluorescence. Each real-time RT-PCR assay consists of unknown samples, including negative extraction controls, and one negative amplification control consisting of nuclease-free water. Positive controls were made with the use of in vitro–synthesized transcripts as controls. The copy number was calculated by using the formula described by Adams that included 104, 103, and 102 copies of genome equivalent (GE) of in vitro–synthesized RNA transcripts. The test was considered positive if there was a typical S-shape amplification curve and the amplification curve, which was detected at *Ct* ≤ 40 for the FAM channel. The result was considered negative (virus was not detected) if there was no typical S-shape amplification with no *Ct* or *Ct* > 40 for FAM.

### 2.5. Full-Length PCR

Synthesis of long cDNA fragments was performed using AddScript cDNA Synthesis Kit (ADD BIO, South Korea) according to the manufacturer's instruction. The full sequence of SARS-CoV-2 from the first patient in the Korean reference sequences was retrieved from the NCBI Reference Sequence Database (https://www.ncbi.nlm.nih.gov/nuccore/NC_045512). To design the primer sets, we utilized the Primer3 tool (http://primer3.wi.mit.edu). The primer sets designed and used in this study were 5′-CATTCGTTTCGGAAGAGACAGG-3′ and 5′-TTAGACCAGAAGATCAGGAACTC-3′. To make a 10 *μ*M working solution of primer, the 100 *μ*M stock solution was diluted (1 : 10) with sterile ddH_2_O. Gradient PCR was carried out using the primer pairs, to optimize the annealing temperature and amplification of targeted fragments using T100 Thermal Cycler (Bio-Rad). The PCR process was performed in a total volume of 50 *μ*l containing 25 *μ*l of Taq DNA Polymerase Master Mix RED2x (Ampliqon, Denmark), 1.5 *μ*l (10 *μ*M) of each primer, 5 *μ*l of cDNA template, and PCR grade water to make up the 50 *μ*l volume. PCR cycling conditions were as follow: one cycle of 95°C for 5 min; 40 cycles of 95°C for 40 s, 58°C for 35 s, 72°C for 45 s, and one cycle of 72°C for 5 min. The amplicons were analyzed by 2% agarose gel electrophoresis [18]. The PCR products were gel-purified using the AccuPrep® PCR/Gel Purification Kit (Bioneer, Korea). Both strands of the PCR products were sequenced by the 1^st^ BASE company (Singapore), using the two forward and reverse primers. Finally, the sequences of the PCR products were compared with known sequences of the E genes of SARS-CoV-2 in the GenBank database.

## 3. Results

**Figure 2:**
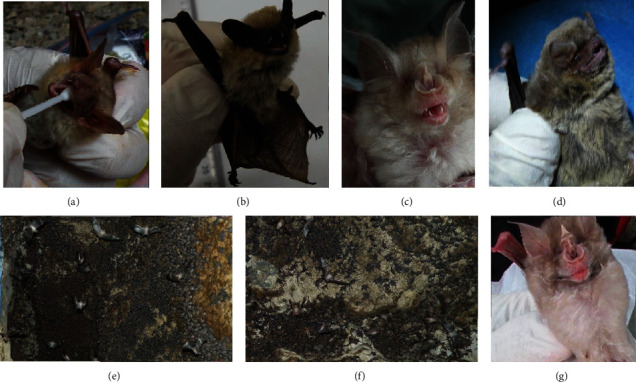
Photos of some individuals or colonies of bats sampled in the present study: (a) *Myotis blythii;* (b) *Hypsugo savii;* (c) *Rhinolophus euryale;* (d) *Miniopterus pallidus;* (e, f) the colony of *Rhinopoma microphyllum;* and (g) *Rhinolophus ferrumequinum*.

Overall, a total of 183 bats were caught from five caves in the south and northwest of Iran. The caught bats belonged to the species of Mediterranean horseshoe bat (*R. euryale*), greater horseshoe bat (*R. ferrumequinum*), Savi's pipistrelle (*Hypsugo savii*), pale bent-wing bat (*Miniopterus pallidus*), lesser mouse-eared bat (*Myotis blythii*), and greater mouse-tailed bat (*Rhinopoma microphyllum*); Figure 2 and Table 1.

**Figure 3:**
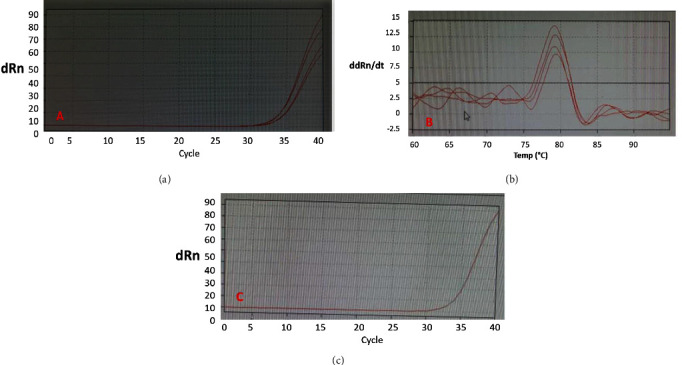
RT-PCR results showing the positive SARS-CoV-2 for alimentary specimens from two Mediterranean horseshoe bats (*R. euryale*): (a) amplification curves of SARS-CoV-2 positive samples derived from bat and positive plasmid control using a SYBR-Green One-Step qPCR assay; (b) melting curve analysis of positive SARS-CoV-2 samples of bat origin; and (c) amplification curves of positive plasmid control.

The E gene of the SARS-CoV-2 was detected in the anal swabs of two bats from Kermanshah, but none of the respiratory samples were positive (Figure 3). The mean *Ct* value detected in the positive samples for SARS-CoV-2 was 30.50 (range 28.27–32.73).

## 4. Discussion

Bats may be carriers of important pathogens, including viruses, to humans. Considering that some viruses can appear specifically in certain populations of bats and also due to the emergence of recombinant viruses, the study of the infection of bats with different viruses is of great importance. SARS-CoV-2 has been shown to have a high nucleotide sequence similarity to a bat SARS-related CoV (bat-SL-CoVZC45, Accession No. MG772933) and only 79.5% genome sequence similarity to SARS-CoV. The virus along with SARS-CoV and bat SARSr-CoVs have been clustered in subgenus Sarbecovirus [19–22]. Genomic analysis of SARS-CoV-2 and four typical coronaviruses (bat SARSr-CoV-Rp3, CoV-ZC45, CoV-ZXC21, and SARS-CoV-Tor2) showed a likelihood of recombination between SARS-CoV-2 and other coronaviruses among the subgenus [19, 23]. Zhou et al. [22] sequenced samples from seven patients and bats, and found that this SARS-CoV-2 shared 96.2% overall genome sequence identity with a bat coronavirus RaTG13 from horseshoe bats (*Rhinolophus*). This finding provided further evidence that SARS-CoV-2 most likely originated from bats [22]. Previously, horseshoe bats were identified as natural hosts for SARS-related coronaviruses which were the direct progenitors for the origin of SARS-CoV [4, 24]. So far, there has been no study related to the infection of Iranian bats with coronaviruses. In the current study, the possible infection of Iranian bats with coronaviruses was assessed for the first time on bats belonging to the family of Rhinolophidae, Vespertilionidae, and Rhinopomatidae. The results showed that alimentary specimens from only two of the Mediterranean horseshoe bats (*R. euryale*) from Kermanshah province located in the northwest of Iran were positive whereas none of the bats' respiratory specimens was positive for the coronaviruses. However, there is a possibility of false negative cases in this study, which should be considered in the interpretation of the results. On the other hand, there is a possibility of false negative cases in this study, which should not be overlooked.

Although, the positive sample results were based on RT-PCR of a 221 bp fragment of the E genes of SARS-CoV-2, but it is not possible to confirm the infection of Iranian bats with cSARS-CoV-2 with high confidence.

## 5. Conclusion

Although, based on the findings of the molecular evaluation, the infection of bats with SARS-CoV-2 was determined in this study, further studies are needed on a larger number of bats, particularly horseshoe bats, to confirm the potential infection of Iranian bats with SARS-CoV-2.

## Figures and Tables

**Table 1:** Bat species captured in the present study and their sampling locations.

Species	Sampling location	Number
*Hypsugo savii*	Fars, Ardakan	1

*Rhinolophus euryale*	Kermanshah province	38

*Rhinolophus ferrumequinum*	Kermanshah province	14

*Miniopterus pallidus*	Fars, Marvdasht	15
	Fars, Tadovan	14
	Fars, Ghader Abad	16
	Kurdistan, Bijar	15

*Rhinopoma microphyllum*	Fars, Tadovan	14

*Myotis blythii*	Fars, Marvdasht	12
	Fars, Tadovan	10
	Fars, Ghader Abad	22
	Kordestan, Bijar	12

## Data Availability

Data used to support the findings of this study are included in the article.
